# Caries pattern and dental treatment features of children with autism spectrum disorder under general anesthesia

**DOI:** 10.1097/MD.0000000000041867

**Published:** 2025-03-21

**Authors:** Chun-Cheng Lai, Chia-Chan Wu

**Affiliations:** a Department of Pediatric Dentistry, Far Eastern Memorial Hospital, Taipei, Taiwan; b Graduate Institute of Clinical Dentistry, School of Dentistry, National Taiwan University, Taipei, Taiwan; c Department of Anaesthesiology, Far Eastern Memorial Hospital, Taipei, Taiwan.

**Keywords:** autism spectrum disorder, behavior, dental caries, general anesthesia

## Abstract

Difficulty in the cooperation of children with autism spectrum disorder (ASD) leads to a poor diagnosis of caries patterns and poor clinical and radiographic findings. This study aimed to investigate the association between caries patterns and treatment characteristics in children with ASD and compare these variables with those in healthy children treated under general anesthesia. The medical records of children with ASD undergoing dental treatment under general anesthesia (n = 40) were analyzed in this study. The collected data included demographic profile, medical status (ASD severity and associated comorbidities), dental history (behavior, care approach, past dental treatment, and follow-up period), caries pattern (surface and depth), and clinical and radiographic findings (e.g., percussion/palpation pain, fistula, furcation involvement, and pathologic resorption). We compared the caries patterns and treatment of children with ASD to those of their healthy counterparts (n = 40). In children with ASD, age and severity were significantly associated with presentation behavior (*P* = .03 and *P* = .04) and the chosen care approach (*P* = .03). Clinical and radiographic examinations revealed that furcation involvement or pathological resorption was more frequent in children with ASD from families with lower average monthly income (*P* = .05) and in those with associated comorbidities (*P* = .02). Caries involving 1 or 2 surfaces were more prevalent in children with ASD, with a significant proportion extending to the dental pulp (*P* < .001). Dental caries characteristics and patterns among children with ASD differ from those of healthy children, which influences dental treatment decisions.

## 1. Introduction

Autistic spectrum disorders (ASD) are a heterogeneous group of neurodevelopmental conditions, including autism, Asperger syndrome, and pervasive developmental disorders not otherwise specified.^[1]^ The prevalence in the United States is reportedly 2.5%, while in Taiwan, ASD accounts for 1.0%–2.0% of the total population.^[2,3]^ The 3 core characteristics of ASDs are social deficits, communication impairment, and restrictive and repetitive interests. ASD onset occurs during childhood, with signs detectable as early as 18 months of age.^[4]^ Children with ASD may also have comorbidities such as attention deficit hyperactivity disorder (ADHD), epilepsy, anxiety, sleep disorders, and depressive disorders, making the diagnosis of ASD more challenging.^[5]^

Poor oral hygiene and gingivitis compromising periodontal health are common findings in children with ASD.^[6]^ Hence, there is an urgent need to maintain periodontal health. However, the reported oral health status and dental caries experience among children with ASD have been conflicting.^[7,8]^ According to the caries-risk assessment tool of the American Academy of Pediatric Dentistry, these children have particular care needs and are considered at high risk of dental caries.^[9]^ In contrast, some studies have reported that people with ASD tend to be caries-free and have lower decayed, missing, and filled teeth (DMFT) scores.^[9,10]^

The severity of dental caries in children with ASD is partially based on their degree of difficulty in seeking professional care. Children with ASD have unmet dental needs, poor communication and social skills, and a highly sensitive personality.^[1]^ Professional behavioral guidance is needed when managing children with ASD. However, some dental practitioners have inadequate training in delivering care to patients with special needs. As a result, dental treatment under general anesthesia or sedation is suggested, even when dental caries are not severe.^[11,12]^ Treatment under general anesthesia may hinder future treatment owing to augmented expenses.

These barriers have led to differences in caries characteristics and treatment experiences between children with ASD and their healthy counterparts. Arnold et al reported that children with ASD received significantly fewer pulpotomies and crowns than healthy children under general anesthesia (GA).^[13]^ To date, objective assessments of patient characteristics, including demographic factors and behavior at presentation, dental treatment, care approach and follow-up periods, in children with ASD have been inadequately evaluated. Notably, the caries pattern, including affected surfaces, depth, and radiographic findings, has not been previously reported in children with ASD, possibly because of their poor cooperative ability. This study aimed to evaluate the dental caries characteristics and treatment among children with ASD and compare them with those of healthy children who underwent dental treatment under general anesthesia.

## 2. Materials and methods

### 2.1. Subjects and ethical approval

This study included 40 children with ASD and 40 healthy controls at the Pediatric Dental Department of Far Eastern Memorial Hospital, Taipei, Taiwan, from July 1, 2020, to December 31, 2023. Data from all patients with ASD (up to 15 years of age) were collected, including sex, age, economic level, and health insurance coverage. According to previously published studies, the actual number of children with ASD may not exceed 2.0%.^[3]^ With 5.0% precision error and 5.0% type I error, the required sample size was calculated as 30 individuals for each main study group. To compensate for the missing data, the sample size was increased to 80 individuals. All patients included in this study had an American Society of Anesthesiologists classification of either 1 or 2. The study complied with the Declaration of Helsinki on medical protocol, and the research ethics review committee of the Far Eastern Memorial Hospital approved the study. Caregivers of the patients were verbally contacted. Informed consent was not provided because the procedures were conventional therapies and had been completed before research. Participant anonymity was maintained during and after data collection by using code names.

The clinical diagnosis of ASD includes the following core symptoms: social deficits, communication impairment, and restrictive and repetitive interest. To further investigate the severity of ASD, the Diagnostic and Statistical Manual of Mental Disorders-5 classification of the American Psychiatric Association was considered, including mild level as “requiring support,” moderate level as “requiring substantial support,” and severe level as “requiring very substantial support.”^[7]^

### 2.2. Data collection

Demographic data, medical status, and dental history of healthy children and children with ASD were collected and analyzed. Demographic variables included sex, age (preschooler: younger than 6 years; schoolchild: ≥6 years), economic level (below or above the average monthly household income OF 2920 USD), frequency of tooth brushing (less than twice or at least twice daily), and health insurance coverage (yes or no). Medical status encompassed ASD severity (mild, moderate, or severe) and presence of comorbidities (yes or no). Dental history, behavior at presentation (assessed using the Frankl behavior rating scale^[14]^: 1 = definitely negative behavior [- -], 2 = negative behavior [-], 3 = positive behavior [+], and 4 = definitely positive behavior [+ +]), care approach at the hospital (full-mouth treatment under GA or simple treatment under physical restraint with local anesthesia (LA) due to new caries after GA), types of dental treatment (restorations, crowns, pulp therapy, or extractions), and follow-up period (<1 year, 1–2 years, or >2 years) were collected. The follow-up period was calculated from the date of treatment under GA to the most recent dental examination. The date of the first treatment under LA for new caries was also recorded. To mitigate the risks associated with GA, no further GA treatment was administered for new caries after the initial surgery. Children with ASD were matched with 40 healthy counterparts treated with GA as the control group. The data collected for the control group were the same, except for medical status. GA was administered by a professional anesthesiologist via intranasal intubation. The patient was discharged with a clear consciousness and no adverse events.

### 2.3. Clinical and radiographic examinations

Clinical and radiographic examinations were conducted when the patients first visited our clinic. The children were behaviorally guided using tell-show-do and desensitization procedures. The number and surface of dental caries (1, 2, 3, more than 3), percussion/palpation pain (yes or no), and fistulas (yes or no) were recorded. After the clinical examination, radiographic examination was conducted to finalize the treatment plan. Full-mouth periapical films were used to assess caries depth (enamel, dentin, and pulp), furcation involvement (yes or no), and pathological resorption (yes or no). Owing to limited cooperative ability, passive physical restraint was used with the caregiver’s consent for some patients. Clinical and radiographic findings were independently confirmed by 2 dentists. The dentists were calibrated for the examination and diagnosis of caries in 10 healthy children treated with GA and were not included in this study. In cases of equivocality, the results were assessed by a third pediatric dentist.

### 2.4. Statistical analysis

Concordance between the 2 pediatric dentists was 93.3%. Krippendorff α value from the inter-rater comparison analysis was 0.89. Simple random sampling was used to avoid selection bias. The variables of the patients with missing data were not calculated or assessed. All *P* values were based on 2-tailed statistical analyses. Fisher exact test was performed for categorical variables in small samples (>20% of cells with frequencies <5). Student *t* test, Mann–Whitney *U* test, and Kruskal–Wallis test were used for continuous variables. Subgroup analyses were performed using paired comparisons with Bonferroni correction. Statistical significance was set at *P* *<* .05. The Statistical Program for Social Science Software 20.0 software (IBM Corp., Armonk, NY) was used for all statistical calculations.

## 3. Results

Demographic data, medical status, and dental history are summarized in Table 1, with no missing information identified during the review. Demographic data, dental history, and clinical/radiographic findings were comparable between patients with ASD and healthy children, except for economic background. Most patients with ASD (60.0%) had a monthly household income below average. In comparison, the healthy counterparts (67.5%) had incomes above average (*P* = .02). The study included 40 children with ASD and 40 healthy children, with 31 (77.5%) males and 9 (22.5%) females in each group. Ages ranged from 3 to 15 years for the ASD group and from 3 to 10 years for the healthy group. Among the children with ASD, 72.5%, 22.5%, and 5.0% had mild, moderate, and severe ASD, respectively. All the participants had health insurance coverage. Most children with ASD were preschoolers (75.0%) and 52.5% brushed their teeth at least twice daily. The most frequently reported comorbidities were ADHD (62.5%) and epilepsy/seizures (30.5%). Negative behaviors during hospital visits were observed in 77.5% of the children with ASD. Most (65.0%) patients underwent dental treatment under GA, whereas 35.0% with simpler treatment needs were managed using LA with protective stabilization, often due to new caries after GA. The follow-up period was less than a year in 55.0% of the children with ASD. Crowns, pulp therapy, and restorations were the most common treatment procedures (97.5%, 92.5%, and 77.5%, respectively), and tooth extraction was the least common (35.0%).

**Table 1:** Characteristics of children with autistic spectrum disorder and healthy controls (n = 40 per group, n (%)).

Demographic data*				
		ASD	Healthy	*P* value
Sex	Boy	31 (77.5)	31 (77.5)	>.99
	Girl	9 (22.5)	9 (22.5)	
Age	Preschooler	30 (75.0)	26 (65.0)	.46
	Schoolchild	10 (25.0)	14 (35.0)	
Economic level	Below the average	24 (60.0)	13 (32.5)	.02**
	Above the average	16 (40.0)	27 (67.5)	
Brushing times	Less than twice	19 (47.5)	16 (40.0)	.65
	At least twice	21 (52.5)	24 (60.0)	
Medical status				
ASD severity	Mild	29 (72.5)	nil	nil
	Moderate	9 (22.5)	nil	
	Severe	2 (5.0)	nil	
Associated comorbidities		25 (62.5)	nil	
Dental history*				
Behavior at presentation (Frankel rating scale)	Rating 1 (- -)	21 (52.5)	12 (30.0)	.11
	Rating 2 (-)	10 (25.0)	16 (40.0)	
	Rating 3 (+)	9 (22.5)	12 (30.0)	
	Rating 4 (+ +)	0 (0.0)	0 (0.0)	
Care approach at the hospital	Treatment under GA	26 (65.0)	32 (80.0)	.21
	Treatment under GA and LA	14 (35.0)	8 (20.0)	
Dental procedures at the hospital	Restorations	31 (77.5)	29 (72.5)	.80
	Crowns	39 (97.5)	40 (100.0)	>.99
	Pulp therapy	37 (92.5)	40 (100.0)	.24
	Extractions	14 (35.0)	10 (25.0)	.46
Follow-up period	<1 yr	22 (55.0)	23 (57.5)	.64
	1–2 yr	5 (12.5)	7 (17.5)	
	>2 yr	13 (32.5)	10 (25.0)	
Clinical/radiographic findings*				
Symptoms and sign	Percussion/palpation pain	9 (22.5)	10 (25.0)	>.99
	Fistula	9 (22.5)	4 (10.0)	.23
	Furcational involvement	11 (27.5)	13 (32.5)	.81
	Pathologic resorption	9 (22.5)	7 (17.5)	.78

* The variables were analyzed using Fisher exact test.

** Statistically significant.

The average follow-up periods for children treated with GA and LA in the ASD and healthy groups were 717.4 days and 876.8 days, respectively. Children treated with GA and LA had significantly longer follow-up periods, potentially due to higher compliance (Table S1, Supplemental Digital Content, http://links.lww.com/MD/O591, *P* < .001). The duration from GA treatment to new caries management with LA did not differ significantly between children with ASD and healthy controls (304.1 days vs 397.9 days, *P* = .39).

Tables 2 and 3 present the associations between demographic data, medical status, behavior at presentation, and care approach in children with ASD and healthy controls. No such association was observed in the healthy group. Among the children with ASD, those with moderate or severe ASD exhibited significantly more negative behavior than those with mild ASD (69.0%, *P* = .04). Moreover, significantly more children with moderate or severe ASD required additional treatment under LA for new caries after full-mouth rehabilitation under GA than those with mild ASD (24.1%). Age was also significantly associated with behavior at presentation (*P* = .03). While 86.7% of preschool-aged children displayed negative behavior, only 50.0% of school-aged children did so.

**Table 2:** Association of the demographic profile and medical status of children with autistic spectrum disorder (n = 40) with behavior and care approach provided.

Variable	Sex* n (%)		Age* n (%)		Economic level* n (%)		Brushing times* n (%)		ASD severity* n (%)		Comorbidities* n (%)	
	Boy	Girl	Preschooler	Schoolchild	Below the average	Above the average	Less than twice	At least twice	Mild	Moderate/severe	Yes	No
Behavior at presentation												
Definitely negative/negative	23 (74.2)	8 (88.9)	26 (86.7)	5 (50.0)	20 (83.3)	11 (68.7)	16 (84.2)	15 (71.4)	20 (69.0)	11 (100.0)	20 (80.0)	11 (73.3)
Positive	8 (25.8)	1 (11.1)	4 (13.3)	5 (50.0)	4 (16.7)	5 (31.3)	3 (15.8)	6 (28.6)	9 (31.0)	0 (0.0)	5 (20.0)	4 (26.7)
*P* value	.70		.03**		.44		.46		.04**		.71	
Care approach at the hospital												
Treatment under GA	20 (64.5)	6 (66.7)	21 (70.0)	5 (50.0)	16 (66.7)	10 (62.5)	12 (63.2)	14 (66.7)	22 (75.9)	4 (36.4)	14 (56.0)	12 (80.0)
Under GA and LA	11 (35.5)	3 (33.3)	9 (30.0)	5 (50.0)	8 (33.3)	6 (37.5)	7 (36.8)	7 (33.3)	7 (24.1)	7 (63.6)	11 (44.0)	3 (20.0)
*P* value	>.99		.28		>.99		>.99		.03**		.18	

* The variables were analyzed using Fisher exact test.

** Statistically significant.

**Table 3:** Association of the demographic profile of healthy children (n = 40) with behavior and care approach provided.

Variable	Sex* n (%)		Age* n (%)		Economic level* n (%)		Brushing times* n (%)	
	Boy	Girl	Preschooler	Schoolchild	Below the average	Above the average	Less than twice	At least twice
Behavior at presentation								
Definitely negative/negative	22 (71.0)	6 (66.7)	20 (76.9)	8 (57.1)	8 (61.5)	20 (74.1)	10 (62.5)	18 (75.0)
Positive	9 (29.0)	3 (33.3)	6 (23.1)	6 (42.9)	5 (38.5)	7 (25.9)	6 (37.5)	6 (25.0)
*P* value	>.99		.28		.48		.49	
Care approach at the hospital								
Treatment under GA	24 (77.4)	8 (88.9)	21 (80.8)	11 (78.6)	10 (76.9)	22 (81.5)	12 (75.0)	20 (83.3)
Under GA and LA	7 (22.6)	1 (11.1)	5 (19.2)	3 (21.4)	3 (23.1)	5 (18.5)	4 (25.0)	4 (16.7)
*P* value	.66		>.99		>.99		.69	

* The variables were analyzed using Fisher exact test.

Tables 4 and 5 summarize the clinical and radiographic findings of the children with ASD and healthy controls based on their demographic profiles. Table 6 details these findings in children with ASD according to their medical status. No statistically significant associations were observed between the clinical and radiographic findings and the demographic profiles of healthy children. Children with ASD from families with a monthly income below the average showed a higher likelihood of radiographic signs suggesting poor prognosis (e.g., furcation involvement or inflammatory root resorption) compared to those from families with incomes above the average (*P* = .05). When the radiographic findings of healthy and ASD children were compared in terms of economic level, a statistically significant difference was also found between the positive radiographic findings in ASD groups and the negative radiographic findings in healthy patients (*P* = .007). Additionally, Pathological radiographic signs were significantly associated with the presence of comorbidities (Table 6; *P* = .02).

**Table 4:** The clinical and radiographic findings of children with autistic spectrum disorder according to their demographic profile.

Symptoms and sign	Sex* n (%)		Age* n (%)		Economic level* n (%)		Brushing times* n (%)	
	Boy	Girl	Preschooler	Schoolchild	Below the average	Above the average	Less than twice	At least twice
Clinical findings (percussion/palpation pain/fistula)								
Positive	11 (35.5)	4 (44.4)	11 (36.7)	4 (40.0)	8 (33.3)	7 (43.8)	9 (47.4)	6 (28.6)
Negative	20 (64.5)	5 (55.6)	19 (63.3)	6 (60.0)	16 (66.7)	9 (56.2)	10 (52.6)	15 (71.4)
*P* value	.71		>.99		.53		.33	
Radiographic findings (furcational involvement/pathologic resorption)								
Positive	15 (48.4)	3 (33.3)	13 (43.3)	5 (50.0)	14 (58.3)	4 (25.0)	8 (42.1)	10 (47.6)
Negative	16 (51.6)	6 (66.7)	17 (56.7)	5 (50.0)	10 (41.7)	12 (75.0)	11 (57.9)	11 (52.4)
*P* value	.48		.73		.05**		.76	

* The variables were analyzed using Fisher exact test.

** Borderline significance.

**Table 5:** The clinical and radiographic findings of healthy children according to their demographic profile.

Symptoms and sign	Sex* n (%)		Age* n (%)		Economic level* n (%)		Brushing times* n (%)	
	Boy	Girl	Preschooler	Schoolchild	Below the average	Above the average	Less than twice	At least twice
Clinical findings (percussion/palpation pain/fistula)								
Positive	9 (29.0)	3 (33.3)	7 (26.9)	5 (35.7)	5 (38.5)	7 (25.9)	3 (18.8)	9 (37.5)
Negative	22 (71.0)	6 (66.7)	19 (73.1)	9 (64.3)	8 (61.5)	20 (74.0)	13 (81.2)	15 (62.5)
*P* value	.70		.72		.48		.30	
Radiographic findings (furcational involvement/pathologic resorption)								
Positive	13 (41.9)	2 (22.2)	11 (42.3)	4 (28.6)	7 (53.8)	8 (29.6)	5 (31.3)	10 (41.7)
Negative	18 (58.1)	7 (77.8)	15 (57.7)	10 (71.4)	6 (46.2)	19 (70.4)	11 (68.7)	14 (58.3)
*P* value	.44		.50		.17		74	

* The variables were analyzed using Fisher exact test.

**Table 6:** The clinical and radiographic findings of children with autistic spectrum disorder according to their medical status.

Symptoms and sign	ASD severity* n (%)		Associated comorbidities* n (%)	
	Mild	Moderate/severe	Yes	No
Clinical findings (percussion/palpation pain/fistula)				
Positive	10 (34.5)	5 (45.5)	11 (44.0)	4 (26.7)
Negative	19 (65.5)	6 (54.5)	14 (56.0)	11 (73.3)
*P* value	.72		.33	
Radiographic findings (furcational involvement/pathologic resorption)				
Positive	9 (31.0)	6 (54.5)	13 (52.0)	2 (13.3)
Negative	20 (69.0)	5 (45.5)	12 (48.0)	13 (86.7)
*P* value	.27		.02**	

* The variables were analyzed using Fisher exact test.

** Statistically significant.

**Figure 1.**
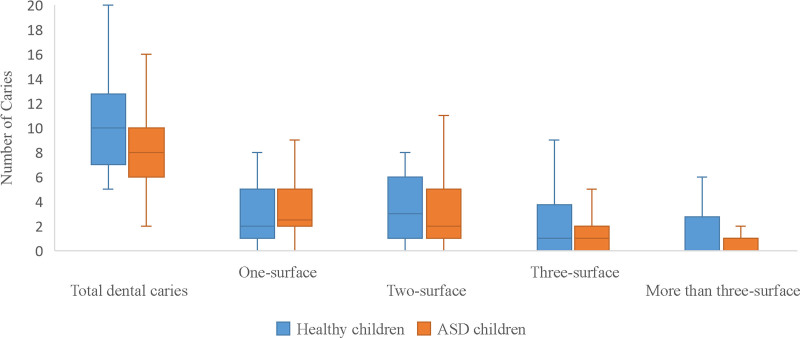
Comparison of the surfaces of dental caries between children with autism spectrum disorder and healthy children using Student *t* test.

**Figure 2.**
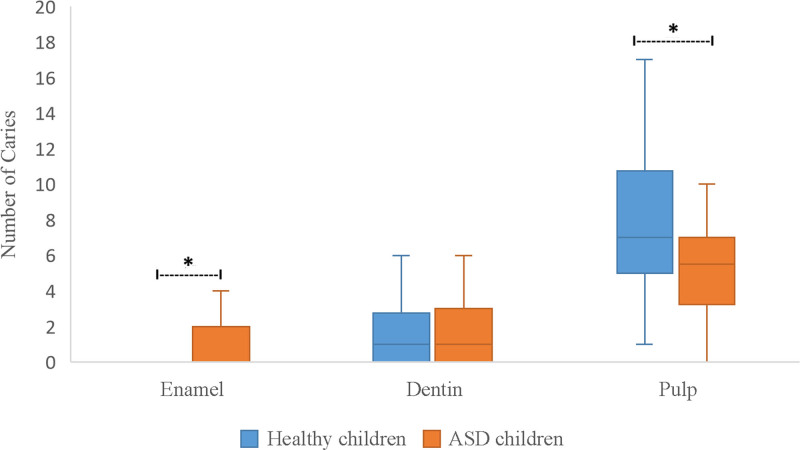
Comparison of the depth of dental caries between children with autism spectrum disorder and healthy children. *Statistically significant.

**Figure 3.**
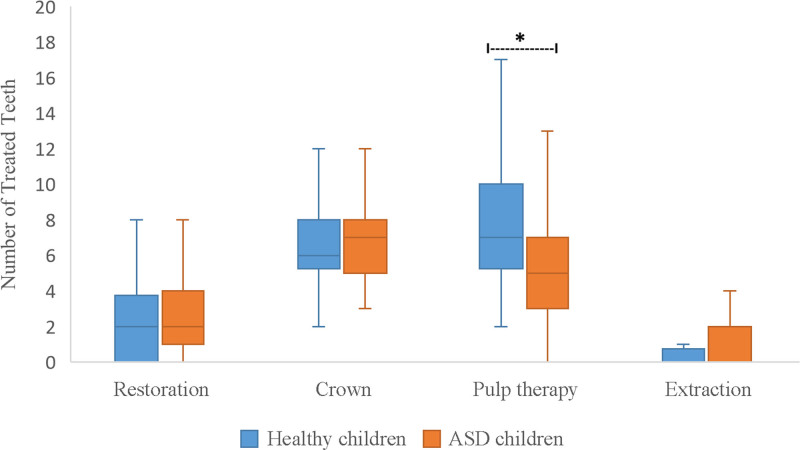
Differentiation of the dental procedures under general anesthesia between children with autism spectrum disorder and healthy children. *Statistically significant.

The number of tooth surfaces affected by dental caries varied significantly between children with mild ASD and those with moderate/severe ASD (Table 7; *P* < .001 and *P* = .02). Paired comparisons revealed that 1- and 2-surface caries were more common in children with mild ASD. In contrast, 1-surface carious lesions were more common in children with moderate/severe ASD than in those with more than 3 surfaces (*P* = .003). Table 8 illustrates the relationship between ASD severity and the depth of dental caries. In children with mild ASD, dental caries involving the pulp were more common than lesions limited to enamel or dentin (*P* < .001). Similarly, in children with moderate/severe ASD, caries extending to the pulp were significantly more frequent than those confined to enamel (*P* < .001). When the number of surfaces and depth of dental caries were compared between children with ASD and healthy controls, there was no difference in the number of carious tooth surfaces (Fig. 1). However, dental carious lesions involving the pulp were more prevalent in the healthy children (*P* = .04). Moreover, a greater number of dental caries extended to the enamel in children with ASD (*P* = .008) (Fig. 2). Figure 3 shows a comparison of the dental treatments. Compared to healthy controls, the number of teeth treated with restorations, crowns, and extractions was similar, but children with ASD had significantly fewer pulp-treated teeth (*P* = .01).

**Table 7:** Association between the severity of autistic spectrum disorder and the invaded surfaces of caries.

ASD severity	Surfaces (median, IQR, number of teeth)				*P* value*
	One^a^	Two^b^	Three^c^	More than three^d^	
Mild	2 (1–4)	1 (0–3)	1 (0–2)	0 (0–1)	<.001**
					a vs c:.006** a vs d: <.001** b vs c: <.001** b vs d: <.001**
Moderate/severe	3 (2–5)	2 (1–4)	1 (0–4)	0 (0–3)	.02**
					a vs d: .003**
*P* value*	.19	.35	.42	.70	

Dental caries affecting 1, 2, 3, or more surfaces are denoted as a, b, c, and d, respectively.

* The variables were analyzed using the Kruskal–Wallis test and Mann–Whitney *U* test. Subgroup analyses were performed using paired comparisons with Bonferroni correction.

** Statistically significant.

**Table 8:** Association between the severity of autistic spectrum disorder and the depth of caries.

ASD severity	Depth (median, IQR, number of teeth)			*P* value*
	Enamel^a^	Dentin^b^	Pulp^c^	
Mild	0 (0–2)	1 (0–3)	5 (4–7.5)	<.001**
				a vs c: <.001** b vs c: <.001**
Moderate/severe	0 (0–3)	2 (0–3)	6 (1–7)	.02**
				a vs c: <.001**
*P* value*	.82	.62	.76	

Dental caries extending to enamel, dental, or pulp are denoted as a, b, and c, respectively.

* The variables were analyzed using the Kruskal–Wallis test and Mann–Whitney *U* test. Subgroup analyses were performed using paired comparisons with Bonferroni correction.

** Statistically significant.

## 4. Discussion

In this study, ASD severity and age affected the children’s behavior at presentation. Severe ASD and younger age increased the probability of uncooperative behavior in dental clinics. These factors result in difficulties accessing dentists who are willing to provide treatment to children with ASD. Alshihri et al reported that 37% of mothers reported that most dentists did not know how to manage uncooperative behaviors, and 44% of patients with ASD reported unpleasant past dental experiences.^[1]^ Behavioral guidance to reduce anxiety and fear is highly recommended in children with ASD. Desensitization before commencing dental treatment to familiarize children with the room and staff is important.^[15]^ Sedation or general anesthesia should be considered if appropriate behavioral guidance has been attempted; however, the patient remains uncooperative.

In this study, ASD severity correlated with hospital treatment as a care approach. The probability of treatment with LA after treatment with GA is higher for children with moderate or severe ASD, possibly because of difficulties in maintaining good oral hygiene. In a study by Alghafis,^[12]^ 78.8% of children with moderate ASD and 82.4% with severe ASD reported repeated GA treatment, suggesting a great reliance on dental treatment under GA as the care approach. The effectiveness of nitrous oxide relative analgesia in children with ASD is controversial and failure may be related to a lack of compliance.^[12]^ A previous study demonstrated that 50% nitrous oxide provided a high success rate (88%) in children with mild ASD.^[16]^

Pulpectomy is indicated for primary teeth with furcation involvement due to irreversible pulpitis or pulp necrosis. Moreover, pathologic root resorption may occur because of longstanding infection. In this study, pathological radiographic signs were more prevalent in children with ASD from families with a monthly income below the average and associated comorbidities. These findings imply more plight for patients with these 2 features in seeking dental care.

Family economic level was comparatively lower among children with ASD than among healthy children. Alshihri et al stated that 75.4% of mothers of children with ASD declined dental treatment because of expenses, leading to caries progression over time.^[1]^ Moreover, it is reasonable to assume that the presence of comorbidities increases ASD severity. ADHD is the most frequent comorbidity in ASD. According to a previous epidemiological study, 28% of patients with ASD have comorbid symptoms of ADHD.^[5]^ In 2010, Ronald et al identified ADHD behaviors in 312 2-year-old twins with ASD through an analysis of the child behavior checklist. These autistic traits were slightly positively correlated with ADHD behaviors (*R* = 0.23–0.26).^[17]^ Craig et al (2015) divided 181 patients into 4 diagnostic groups, ADHD, ASD, and ASD + ADHD, and compared the data with a control group to identify clinically distinctive features of different ASD and ADHD phenotypes.^[18]^ Using the Conners’ rating scale-revised, SNAP-IV rating scale, and child behavior checklist, they concluded that the ASD + ADHD group was more likely to have oppositional defiant disorder, cognitive problems, anxiety, perfectionism, social problems, and somatic complaints than the ASD group.

Current evidence regarding the experience of dental caries in children with ASD is conflicting. In this study, 1- and 2-surface caries were more frequently detected in children with mild ASD than in those with at least 3-surface caries. In this study, children with ASD had more caries extending to the enamel and less caries involving the pulp (and thus less pulp therapy) than healthy controls. Our findings support the hypothesis that individuals with ASD are more likely to be caries free. In a study by Vajawat and Deepika, the mean DMFT scores in those with ASD were 1.297 and 3.736, respectively, in the unaffected controls.^[10]^ The prevalence of caries was lower in children with ASD who had a permanent dentition. The incidence of caries increases with age in children with ASD and their healthy counterparts. In the primary dentition, Fakroon et al showed that the mean dmft index of children with ASD was 1.13, and that of the healthy group was 2.85,^[19]^ which implies a lower caries risk among children with ASD, which is comparable with our results.

This study has some limitations. First, no information regarding dietary habits and plaque amount was available. However, dietary factors and the amount of plaque may influence the severity of dental caries and selection of dental procedures. Second, when describing the caries pattern, we focused only on the number of affected teeth, surfaces, and depth. The International Caries Detection and Assessment System may be more appropriate for caries evaluation.^[20]^ Third, the retrospective study design and relatively small sample size from a single hospital may hinder the generalizability of the results. This is the first study to explore the relationship between dental treatment characteristics and caries pattern in children with ASD via full-mouth radiographic examinations. Previous studies have relied only on the DMFT index to assess dental caries, which may lead to underestimation of caries experience. The association between dental treatment characteristics and International Caries Detection and Assessment System scores should be investigated further in children with ASD. Importantly, to improve the long-term health of children with ASD, a framework for personalized caries management should be developed.

## 5. Conclusion

Severity and age have an impact on behavior at presentation in children with ASD. In turn, severity affected the care approach and treatment provided.

Radiographic examination findings suggest that low socioeconomic background and comorbidities among children with ASD are 2 factors that influence furcation involvement or pathologic resorption. In children with ASD, dental caries tends to involve 1 or 2 surfaces and may extend close to the dental pulp.

Compared with healthy controls, children with ASD had more caries limited to the enamel and fewer carious lesions involving the pulp, leading to a lower need for endodontic therapy.

## Acknowledgements

The authors wish to appreciate the help of all faculty members of the Department of Dentistry at the Far Eastern Memorial Hospital and the consultation on biological statistics from Prof Yung Kai Huang.

## Author contributions

**Conceptualization:** Chun-Cheng Lai.

**Data curation:** Chun-Cheng Lai, Chia-Chan Wu.

**Formal analysis:** Chun-Cheng Lai.

**Investigation:** Chun-Cheng Lai, Chia-Chan Wu.

**Methodology:** Chun-Cheng Lai, Chia-Chan Wu.

**Project administration:** Chun-Cheng Lai.

**Software:** Chun-Cheng Lai.

**Supervision:** Chun-Cheng Lai, Chia-Chan Wu.

**Validation:** Chun-Cheng Lai, Chia-Chan Wu.

**Visualization:** Chun-Cheng Lai, Chia-Chan Wu.

**Writing – original draft:** Chun-Cheng Lai.

**Writing – review & editing:** Chun-Cheng Lai, Chia-Chan Wu.

## Supplementary Material

SUPPLEMENTARY MATERIAL
