# Supplementary material for: Caries pattern and dental treatment features of children with autism spectrum disorder under general anesthesia
Source: Medicine (Baltimore). 2025 Mar 21;104(12):e41867. doi: 10.1097/MD.0000000000041867 (PMC11936631; doi:10.1097/MD.0000000000041867)
Supplement: SUPPLEMENTARY MATERIAL [file medi-104-e41867-s001.docx]

Supplementary Table 1. Association between the follow-up period (mean ±SD, days) and care approach at the hospital among children with autistic spectrum disorder and healthy control

| **Care approach at the hospital** | **Total Follow-up Period** | | *p* value ^†^ |
| --- | --- | --- | --- |
|  | **Healthy Children** | **Children with ASD** |  |
| Treatment under GA | 319.8  (324.9) | 238.9  (273.9) | .78 |
| Under GA and LA | 876.8  (379.2) | 717.4  (356.5) | .34 |
| *p* value ^†^ | < .001^*^ | < .001^*^ |  |
|  | **Follow-up Period (To the Date of LA)** | |  |
| Under GA and LA | 397.9  (230.3) | 304.1  (245.8) | .39 |

† The variables were analyzed using Student’s t test. * Statistically significant
